# Mass spectrometry-based protein identification by integrating de novo sequencing with database searching

**DOI:** 10.1186/1471-2105-14-S2-S24

**Published:** 2013-01-21

**Authors:** Penghao Wang, Susan R Wilson

**Affiliations:** 1 Prince of Wales Clinical School, University of New South Wales, Australia; 2 Mathematical Sciences Institute, Australian National University, Australia

## Abstract

**Background:**

Mass spectrometry-based protein identification is a very challenging task. The main identification approaches include de novo sequencing and database searching. Both approaches have shortcomings, so an integrative approach has been developed. The integrative approach firstly infers partial peptide sequences, known as tags, directly from tandem spectra through de novo sequencing, and then puts these sequences into a database search to see if a close peptide match can be found. However the current implementation of this integrative approach has several limitations. Firstly, simplistic de novo sequencing is applied and only very short sequence tags are used. Secondly, most integrative methods apply an algorithm similar to BLAST to search for exact sequence matches and do not accommodate sequence errors well. Thirdly, by applying these methods the integrated de novo sequencing makes a limited contribution to the scoring model which is still largely based on database searching.

**Results:**

We have developed a new integrative protein identification method which can integrate de novo sequencing more efficiently into database searching. Evaluated on large real datasets, our method outperforms popular identification methods.

## Background

Mass spectrometry (MS) is a commonly used, high-throughput tool for studying proteins. The procedure of MS-based protein identification involves digesting proteins into peptides, which are then separated, fragmented, ionised, and captured by mass spectrometers. Proteins are finally identified from the peaks of the captured mass spectra using computational methods, where each peak theoretically represents a peptide fragment ion. However accurate identification of proteins from tandem mass spectra is a very challenging task and existing methods can typically identify fewer than 50% of the proteins in a complex sample [1-3]. Therefore, there is a critical need for new protein identification methods that can improve the identification accuracy and reliability.

Existing protein identification methods can be categorised into 2 approaches: the database search approach and the de novo sequencing approach. The database search approach has been widely used and is more popular. It identifies proteins by generating theoretical spectra in silico from a given protein sequence database and comparing experimental spectra with the theoretical ones to find the closest matches. A number of methods have been developed, for example SEQUEST [4] which applies a cross correlation scoring model, X!Tandem [5] which uses a hyper-geometric scoring model, OMSSA [6] which applies a Poisson scoring model, and MASCOT [7] which employs a probability-based scoring model. Despite having the advantage of robustness, the database search approach has several limitations. It is only effective if the proteins of interest are already known and the utilised database contains the correct protein sequences. Unfortunately, this is difficult since many studies involve unknown proteins and protein modifications [8,9]. Therefore, only a portion of the identifications reported by database search methods is correct. In addition, specifying the enzyme used in the proteolytic digestion can also exclude the correct peptides from the database search space and lead to erroneous identifications [10].

The de novo sequencing approach identifies proteins by extracting protein sequence information directly from the spectrum peaks derived from peptide fragment ions without recourse to any protein database. Existing de novo sequencing methods can be classified into two categories. For the first category, such as Sherenga [11] and Lutefisk [12], the problem is projected into graph theory and algorithms used for finding the maximum path in a network topology are applied to achieve identification. In the second category, exemplified by PepNovo [13], probability models for inferring protein sequences from the spectrum peaks are applied. However, the main idea remains the same: to find the longest possible peptide sequence that best matches the experimental spectrum. The de novo sequencing approach is the only feasible means for finding novel proteins, detecting amino acid mutations, and so on. However, de novo sequencing is difficult because tandem mass spectra are inherently deficient [14]. Even if the optimal path can be obtained, it may not always yield the correct peptide sequences because peptide fragment ions are usually under-represented and many intensive peaks in the spectra may derive from various interferences.

An "intermediate" approach has been proposed to integrate the aforementioned two approaches: short peptide sequence fragments or "tags" are inferred directly from the spectrum and a database search is performed to find complete peptide sequences that match the sequence fragments. Thus, the identification process is able to incorporate information from the two heterogeneous approaches. This integrative approach has great potential and several methods have been developed, including GutenTag [15], Inspect [16], MultiTag [17], etc. These methods perform favourably compared to existing database search and de novo sequencing methods. However, current implementation of this integrative approach has several limitations. Firstly, the utilised de novo sequencing mechanisms are rather simplistic. Therefore, the inferred sequence tags are short, and usually consist of 3 amino acid residues. Such small tags only offer limited information and may not significantly improve the accuracy. When the sample is complex, the errors in these tags may increase and lead to incorrect identifications [18]. Secondly, most methods try to find exact sequence matches of the tags to the database. This undermines the identification of new proteins and protein modifications. Even if methods like MultiTag take a step forward to tolerate a couple of mismatches, only marginal improvement can be obtained. Thirdly, existing sequence tag methods still apply database search-centred scoring models to which de novo sequencing makes little contribution. With the introduction of high precision ion trap instruments, this leaves many signal-rich spectra seriously under-utilised.

Therefore, we have developed a new integrative method, NovoDB, for protein identification. The method extends the integrative approach introduced by the sequence tag methods and has several advantages. Firstly, it incorporates a sophisticated de novo sequencing algorithm and infers the peptide sequences in a data-driven manner. Much longer sequence tags can be inferred accurately. Secondly, it does not rely on finding exact sequence tag matches in the database but employs a dynamic programming approach to better tolerate sequencing errors. Thirdly, our method employs a simple scoring model that gives more weight to the de novo sequencing. Evaluated on large datasets, generally our method is able to identify more proteins at the same false discovery rate (FDR) when compared to 3 popular methods, including database search-based X!Tandem, de novo sequencing-based PepNovo, and sequence tag-based GutenTag.

## Methods

**Figure 1:**
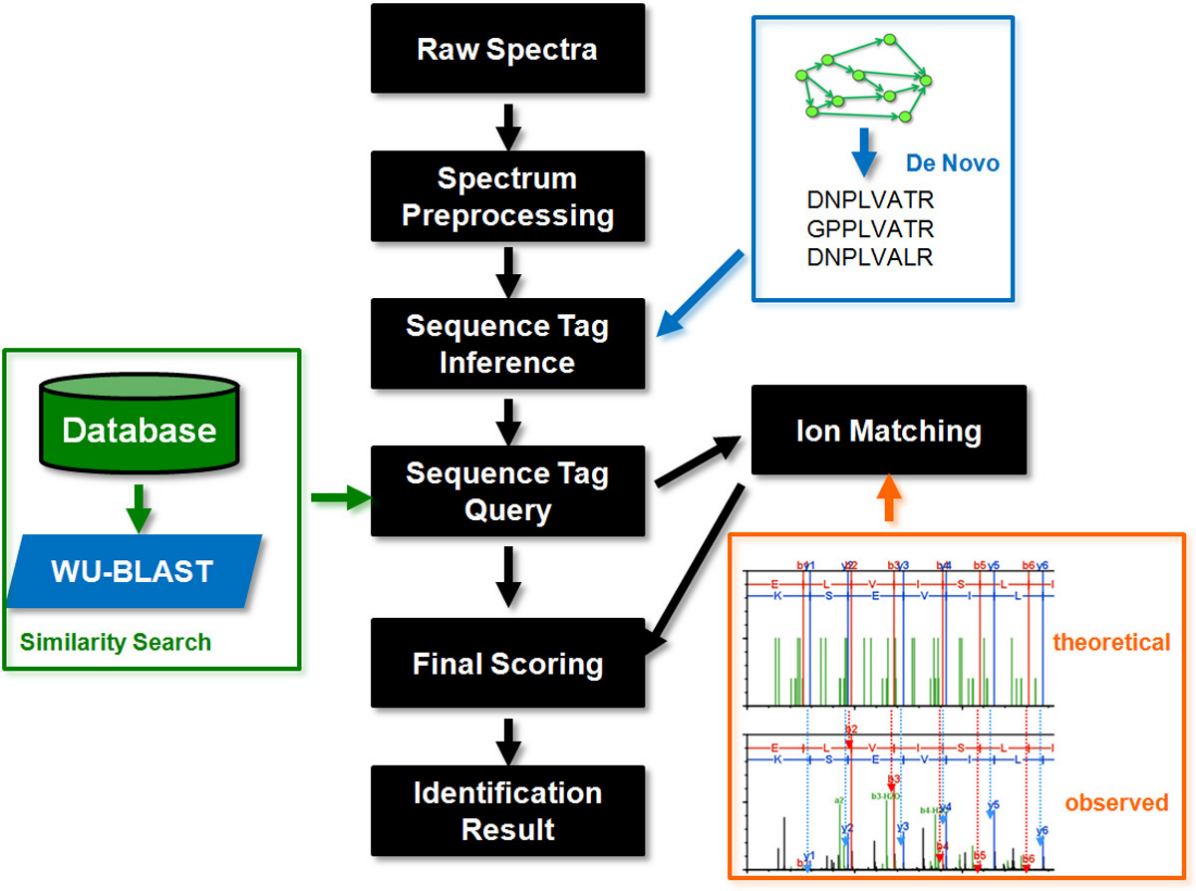
**Brief overview of the NovoDB identification method**.

Our approach has 5 major stages: (1) spectrum preprocessing, (2) sequence tag inference, (3) sequence tag query, (4) spectrum ion match, and (5) final scoring. An overview of the method is given in Figure 1.

### Spectrum preprocessing

The first stage is to preprocess the spectra and normalise the peak intensities. Our method uses two versions of the peak intensities: the continuous intensities and the discrete intensities. For each spectrum, the method firstly determines the baseline intensity and divides each peak's intensity to the baseline so that a normalised intensity is obtained. The continuous intensities are used for the ion matching and the final score calculation, while the discrete intensities are used for the de novo sequencing-based tag inference. The normalised peak intensities are discretised into four levels: no signal, low signal, medium signal, and strong signal. The method removes the low signal peaks by using a sliding window mechanism and discards all the peaks except the top several peaks within each sliding window. Because different regions of the spectrum have different characteristics, our method organises peaks into five regions based on the mass to charge ratio and utilises this information in the sequence tag inference.

### Sequence tag inference

The second stage is to infer a number of peptide sequence tags directly from the spectrum. Instead of inferring short sequence tags which usually leads to misidentifications [18], NovoDB applies a more sophisticated algorithm to dynamically infer longer peptide sequences in a data-driven fashion. This is achieved by incorporating a hybrid de novo sequencing approach which integrates a Bayesian Network probability model with a dynamic programming algorithm to infer the most probable tags [19]. The sequence tag inference stage consists of 3 major steps in total.

#### (1) Spectrum graph construction

Given a preprocessed spectrum *S*, NovoDB builds the spectrum graph and connects all edges if the mass difference between two vertices approximates the residue mass of an amino acid or other mass offsets of a residue derived from ion degradations. Since the most intensive peaks tend to be b- and y-ions, the spectrum graph has vertices for both interpretations. A vertex for an empty peptide and a vertex for the intact peptide are also added. Our method extends the Bayesian Network model used by PepNovo to calculate the probability of observing each vertex of the constructed spectrum graph. The details can be found in [19]. Each vertex of the network contains a conditional probability table given the values of its parent vertices. The probability tables are trained by using the large-scale Seattle dataset [20].

#### (2) Vertex scoring

Each vertex is scored by comparing one hypothesis that the peak is a real fragment ion to the other hypothesis that the match is random. It is calculated by the likelihood ratio:

(1) $$O_{i}\left(m_{j},S\right)=\log\left(\frac{P_{real}\left(t|m_{j},S,R,NT,CT\right)}{P_{random}\left(t|m_{j},S\right)}\right),$$

where *O_i _*represents the score for vertex *i*, *m_j _*is the mass, *S *is the spectrum, *t *is the complete set of all peak intensities of *S*, *R *is the peak region, *NT *represents the N-terminal residue's chemical effect, and *CT *represents the C-terminal residue's chemical effect. Under the hypothesis that the mass matches are random events, the value of the denominator in (1), namely *P_random_*(*t*|*m_j_*, *S*), can be calculated as the product of the probabilities of observing individual peaks at their mass positions. To calculate the numerator in (1), first assume *V *is the set of the vertices in the probability network except the roots, then *V *= {b^+^, y^+^, b^+^-H_2_O, y^2+ ^...}. For each vertex *v *of *V*, *w*(*v*) denotes the assigned intensities of *v*'s parents. *P_real_*(*t_v _*= *i*|*w*(*v*) = {*t*_1_, *t*_2_, ...}) is the probability of detecting intensity *i *at fragment ion *v *given the intensities of its parents. Because vertex *v *is independent, the probability of observing ion fragment intensities of *t *given that the possible cleavage occurred at mass *m_j _*in *S *can be calculated as follows:

$$P_{real}\left(t|m_{j},S\right)=\prod_{v\in V}P_{real}\left(t_{v}|w\left(v\right),m_{j},S,R,NT,CT\right).$$

One advantage of the model is that *P_real _*can distinguish the likely combinations of ions and ion degradations from the unlikely combinations.

#### (3) Sequence tag inference

NovoDB finds several top ranking asymmetric paths as the most probable peptide sequences. The method employs the dynamic programming algorithm proposed in [21] to obtain a set of highly scored peptide sequences by exploring the sub-optimal space from the spectrum graph. There are two reasons. Firstly, a number of vertices on the optimal path may be false positives because it is common that many intensive peaks derive from interferences. Secondly, vertices representing the real fragment ions may not always have the highest score and thus will not be included in the optimal path. It is normal that real fragment ions have low intensities or even cannot be detected. The highly similar segments of the sequences correspond to the fragment ions that are likely to be correctly identified, while the ambiguous segments are where the ions are hardly distinguishable from baseline noise. Given these characteristics, the most likely peptide sequence tags are extracted by adapting a dynamic programming-based algorithm similar to ClustalW [22]. In this case, the introduced "gaps" between the sub-optimal peptide sequences correspond to the ambiguous sections of the tandem mass spectrum. Thus, it is able to dynamically generate longer sequence tags than 3 amino acid residues.

### Sequence tag query

After sequence tags are obtained, the next stage is to query a database to see if matches can be found. This is important firstly because the information provided by the database can fill the gaps that de novo sequencing leaves out. Secondly, the sequences directly inferred from the spectrum may not be sufficient to uniquely identify a protein. Thirdly, even though the sequences of a novel protein are not present in the database, homologue proteins may have been discovered and they provide crucial information for validating and understanding the novel protein.

Our method applies a sequence similarity search based on a tailored WU-BLAST algorithm [23]. The algorithm produces error-tolerant scores and does not require long and identical sequences to produce a confident protein hit. The sequence tag query algorithm identifies all high scoring pairs of regions having high local sequence similarities, namely between an individual peptide's sequences in the query and a protein's sequences from the database. We have introduced several modifications to the BLOSSUM62 matrix to suit the sequence query in the context of mass spectrometry. Scores for the two pairs of isobaric amino acid residues: glutamine and lysine, leucine and isoleucine, are substituted for their average values. The specificity of trypsin is considered by reserving the K symbol for the C-terminal lysine and by introducing a new value averaged between arginine and lysine to represent a cleavage site preceding the peptide sequence. Undefined amino acid residues are introduced with zero scores in order to increase the similarity score if peptide sequence tags are incomplete and contain errors. NovoDB ranks the reported peptide hits by similarity scores *S_s _*and constrains the total number of query hits.

### Ion matching

The ion matching stage is based on the dot-product between the observed ions and the theoretical ions generated in silico from the sequence database. Firstly, each peptide hit from the database query will be theoretically fragmented and a vector of peaks *P *will be created representing all possible fragment ions. The intensity of each theoretical peak *P_i _*will be generated using an empirical fragmentation model similar to SEQUEST. In total, there will be 9 types of ions to be modelled. The b^+ ^and y^+ ^ions will be generated having intensities of 100, while a^+ ^ions will have intensities of 20. The b^2+ ^and y^2+ ^ions will have intensities of 50. The b^+^-H_2_O, y^+^-H_2_O, b^+^-NH_3_, and y^+^-NH_3 _ions will all have intensities of 20. Secondly, experimental peaks will be aligned to theoretical peaks and the unmatched peaks excluded from the analysis. The experimental ion series is represented as a vector *I*, where the value of *I_i _*corresponds to the intensity of an observed fragment ion or 0 if no fragment ion is observed. The correlation score *S_c _*is calculated in a similar way to X!Tandem:

$$S_{c}=N_{b}!*N_{y}!*\sum_{i=0}^{n}I_{i}*P_{i},$$

where *N_b _*and *N_y _*represents the number of assigned b- and y-ions respectively. The ion matching score as given assumes an underlying hyper-geometric distribution for a valid match. This model has been shown to be very effective [24]. The ion matching score is calculated for every candidate protein returned by the database query.

### Final scoring

The final identification score *S *is calculated as follows:

$$S=W_{denovo}S_{s}*\left(1-W_{denovo}\right)S_{c},$$

where *W_denovo _*represents the weight. The protein with the highest score is considered to be correct. The delta score *D *is also calculated measuring how good the identification score, *S_max_*, is relative to the second best, namely *S_2nd_*:

$$D=\frac{S_{\max}-S_{2nd}}{S_{\max}}.$$

*S_c_*, *S_s _*and inferred sequence tags are also reported in the final output.

## Results

### Evaluation strategy

#### Datasets

To evaluate the performance of our method, we use the raw spectra from two large-scale datasets as a benchmark: (1) the Aurum dataset [25] and (2) the CPTAC dataset [26] from Clinical Proteomic Technologies Assessment for Cancer. The Aurum dataset is generated from a mixture of 246 known human proteins. The CPTAC dataset comes from a large-scale study of the reproducibility and repeatability of the Universal Proteomics Standard Set 1 (UPS1).

#### Compared methods

We compare NovoDB with 3 other widely used algorithms: (1) the de novo sequencing method PepNovo, (2) the database search method X!Tandem, and (3) the sequence tag method GutenTag. PepNovo is one of the most widely used de novo sequencing methods. X!Tandem has been shown to outperform commercial SEQUEST and MASCOT database search engines on some data [27]. GutenTag has been used as a benchmark for evaluating sequence tag-based methods [17].

#### Evaluation criteria

**Figure 2:**
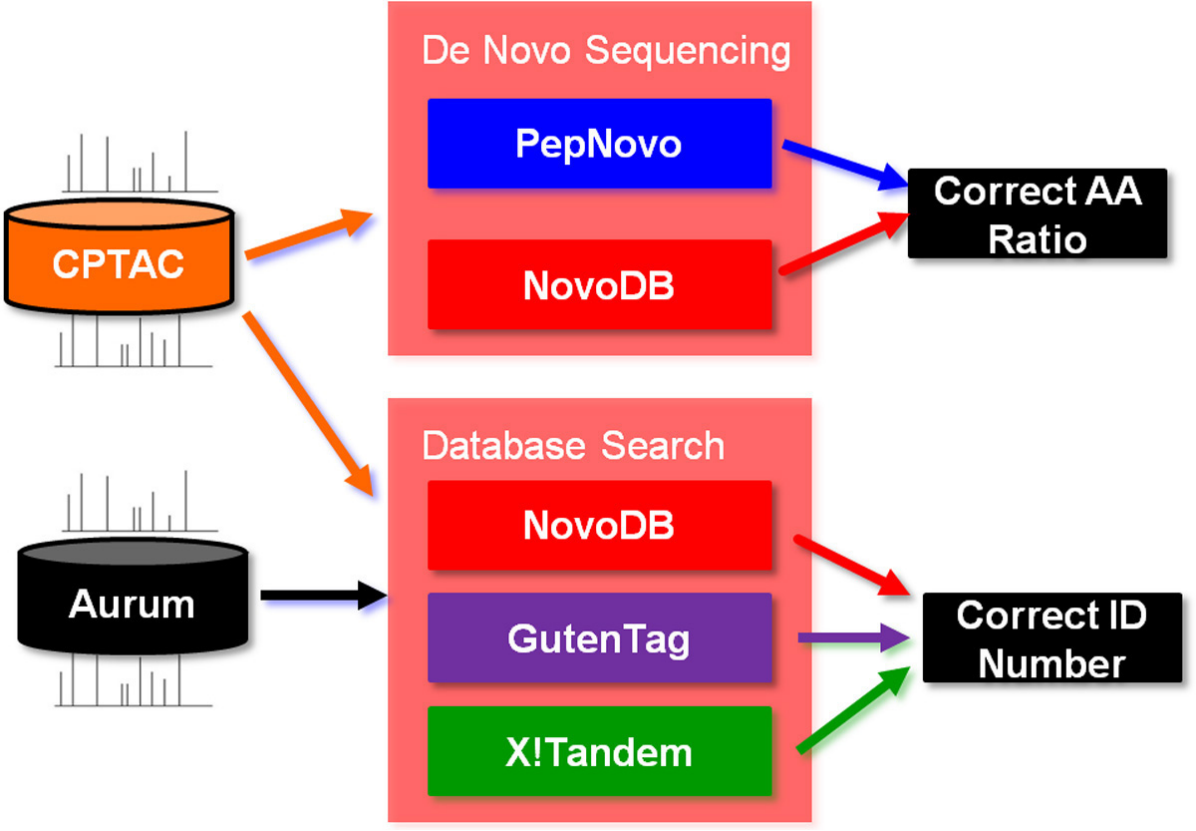
**Overview of the evaluation strategy**.

We use two different performance criteria. Firstly we compare the tag inference results of NovoDB with PepNovo by using the sequence inference accuracy. It is defined as the ratio of the number of correctly identified amino acid residues to the total number of identified residues of a peptide. Secondly, we evaluate how many peptides can be correctly identified by X!Tandem, GutenTag, and NovoDB at the same identification FDR. See Figure 2 for an overview.

### Evaluation results

**Figure 3:**
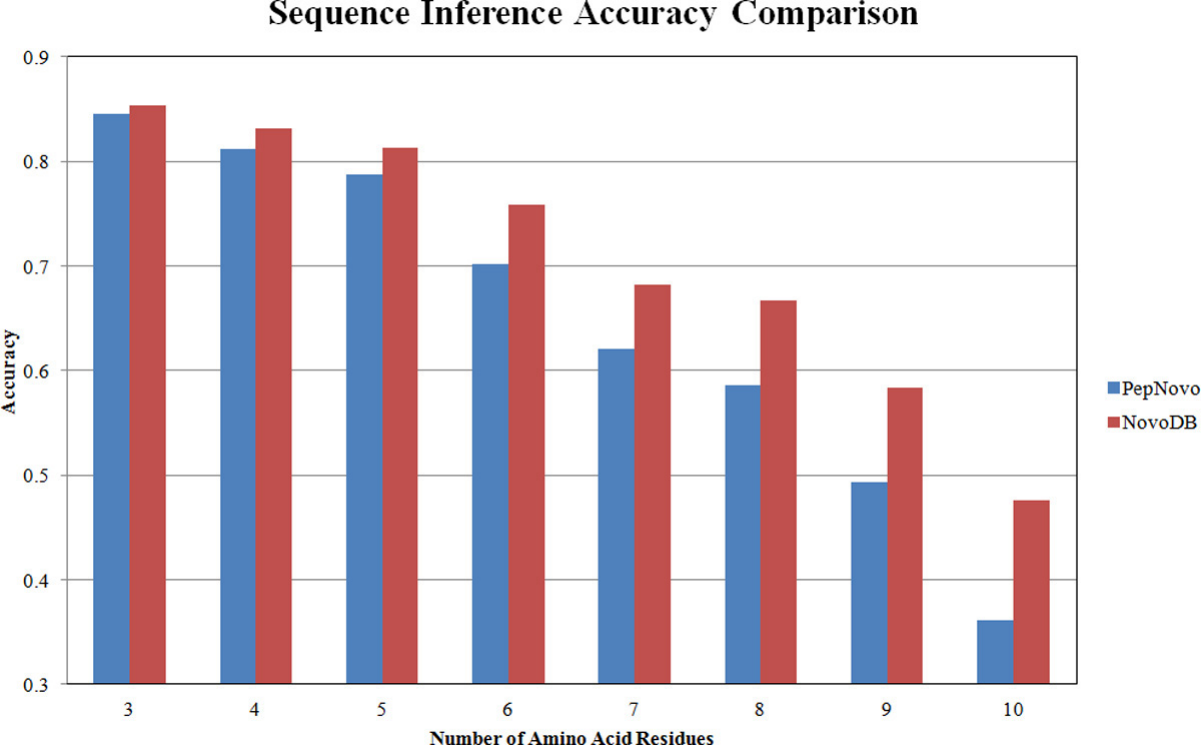
**Results of sequence inference accuracy comparison on CPTAC**.

**Figure 4:**
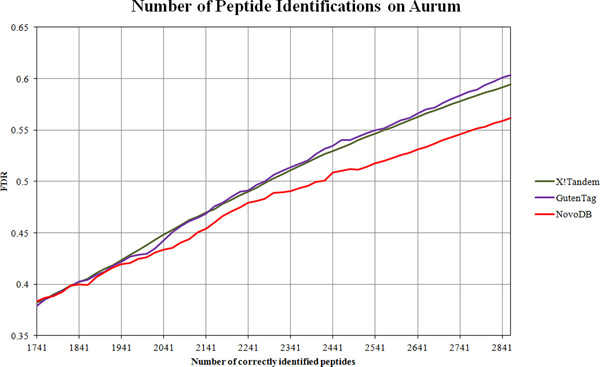
**Results of peptide identification accuracy comparison on Aurum**.

**Figure 5:**
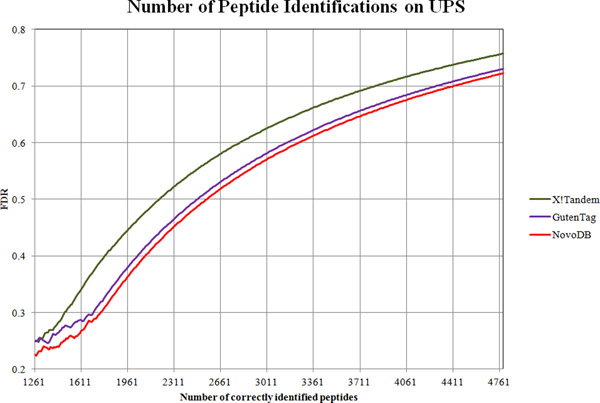
**Results of peptide identification accuracy comparison on CPTAC**.

The overall result of the comparison between PepNovo and NovoDB is given in Figure 3. On average, NovoDB achieves 10% higher accuracy than PepNovo. As expected, NovoDB has much better accuracy in identifying longer peptide sequences. When inferring 5 amino acid residues, NovoDB achieves slightly better accuracy than PepNovo; however when inferring 10 residues, NovoDB doubles the accuracy. This is very important because the length of sequence tags greatly affects the final identification accuracy. Figure 3 demonstrates that both methods can achieve over 80% accuracy when inferring 3 or 4 residues, while the accuracy drops to around 60% for NovoDB and 50% for PepNovo when inferring 9 residues. Therefore, it is clear that one has to keep a balance: it may become detrimental to integrate sequence tags more than 8 residues long, although in theory it is better to incorporate longer tags. The comparison results of X!Tandem, GutenTag and NovoDB are given in Figures 4 and 5. For the Aurum dataset, X!Tandem marginally outperforms GutenTag. This may be due to the complicated spectra of the Aurum dataset. Because only short tags are targeted, it may become difficult for GutenTag to accurately generate a series of non-conflicting sequence tags. On the other hand, the CPTAC dataset was generated on more advanced instruments, so the spectra have higher mass accuracy. This enables GutenTag to more accurately infer short tags. As a result, for a fixed FDR, GutenTag identifies around 12% more peptides than X!Tandem. NovoDB performs significantly better than both methods. On the Aurum dataset, NovoDB increases the identification accuracy by around 13%, while on the CPTAC dataset NovoDB correctly identifies around 16% more peptides than X!Tandem at the same FDR. The performance gap between NovoDB and GutenTag is smaller on the CPTAC dataset. This shows that integrated de novo sequencing has a strong effect on the final results especially when spectra are of good quality. On the other hand, this also indicates that short sequence tags may not be sufficient when the sample contains a large number of proteins and the spectra are of higher complexity.

## Discussion

Sequences obtained by de novo sequencing are valuable and can significantly increase identification coverage when effectively integrated with database searching. With current fast development of new instruments, this becomes crucial because the identification coverage of database search methods cannot be significantly improved with the increasing resolution of the spectra. This serious bottleneck may be due to the reliance on databases, which are seldom complete. On the other hand, the performance of the de novo sequencing approach increases proportionally to the increase of the spectra resolution. In recent years, proteomics research has shifted from a macro qualitative analysis into a micro perspective including the study of glycolysis and phosphoralytion. Unfortunately, database search methods may lead to misidentifications for these applications [3,12]. De novo sequencing remains the only feasible approach in this situation. Therefore, it is essential to integrate de novo sequencing into database searching.

The evaluation results indicate that one should be careful in choosing the length of peptide sequence tags. Feeding longer tags will facilitate database searching and potentially increase the identification coverage. However this may lead to more sequence errors. Existing tag-based methods choose to use very short sequence tags, e.g. 3 residues long. Such an approach may perform well when the spectrum has high signal-to-noise ratio or the protein composition is simple. This explains why GutenTag performs much better on the CPTAC dataset. However, when the spectra are complicated, it becomes difficult for this approach to succeed. Therefore, it is critical to dynamically choose a proper sequence tag length based on each individual spectrum. Results demonstrate that this may be achieved by exploring the top ranking sub-optimal solutions of the spectrum graph. Based on our evaluation, sequence tags of 6 to 7 residues seem to yield the best results. This should be studied further.

How to effectively integrate de novo sequencing and database searching into a single scoring model is an open question. By using existing integrative methods, the incorporated de novo sequencing algorithm is normally simplistic and cannot contribute directly to the score calculation. Given poor quality spectra, this method is quite reliable. However, such a design cannot efficiently utilise the high precision and high resolution provided by the new instruments and may lead to sub-optimal results. It is therefore important to incorporate a sophisticated de novo sequencing algorithm and a more global scoring model that can give de novo sequencing more weight. Based on our evaluation, when the de novo sequencing component can directly contribute to the score calculation, a simple scoring model, as presented by our NovoDB approach, may work well. In theory, it is very desirable to incorporate more advanced scoring models that can integrate more effectively the de novo sequencing component with the database search component. A more advanced scoring model may further improve the identification accuracy. However, there is always a trade-off between the complexity of the scoring model and the computational cost. Therefore, one has to keep a good balance between the two when designing new scoring models. The design of more advanced scoring models is a very interesting direction for future research.

## Conclusions

Protein identification plays a key role in mass spectrometry-based protein research. Existing protein identification methods have limitations which usually lead to low identification coverage. We have developed a new integrative protein identification method which can integrate de novo sequencing more efficiently into database searching. Evaluated on large real datasets, our method outperforms popular identification methods. This performance demonstrates that in order to significantly improve the identification coverage and accuracy, it may be necessary to integrate effectively heterogeneous approaches into protein identification.

## Competing interests

The authors declare that they have no competing interests.

## Authors' contributions

PW and SRW conceived the idea and contributed equally to the work. PW implemented the system and performed the experiments. PW and SRW wrote the manuscript together. All authors read and approved the final manuscript.

## Declarations

The publication of this article was funded by the Australian National Health and Medical Research Council (NHMRC) grant 525453.

This article has been published as part of *BMC Bioinformatics *Volume 14 Supplement 2, 2013: Selected articles from the Eleventh Asia Pacific Bioinformatics Conference (APBC 2013): Bioinformatics. The full contents of the supplement are available online at http://www.biomedcentral.com/bmcbioinformatics/supplements/14/S2.
